# Intrathoracic amyloid tumors that presented as yellowish multinodular endobronchial protrusions with irregular vascularity and easy bleeding

**DOI:** 10.1111/1759-7714.13159

**Published:** 2019-08-04

**Authors:** Hiromi Tomono, Hiroshi Soda, Yuichi Fukuda, Yasuhiro Tanaka, Sawana Ono, Midori Shimada, Keisuke Iwasaki, Masashi Hisanaga, Hiroyuki Yamaguchi, Hiroshi Mukae

**Affiliations:** ^1^ Department of Respiratory Medicine Sasebo City General Hospital Nagasaki Japan; ^2^ Department of Pathology Sasebo City General Hospital Nagasaki Japan; ^3^ Department of Otolaryngology Sasebo City General Hospital Nagasaki Japan; ^4^ Department of Respiratory Medicine Nagasaki University Graduate School of Biomedical Sciences Nagasaki Japan

**Keywords:** Amyloidosis, bleeding, bronchoscopy, narrow band imaging

## Abstract

Immunoglobulin light‐chain (AL) amyloidosis is a monoclonal plasma cell neoplasm that has a tendency to bleed easily. However, the potential risks of transbronchial biopsy in such cases have not been fully proven. Here, we report a case of parotid and intrathoracic AL amyloid tumors that presented as endobronchial protrusions that bled easily. Bronchoscopy under conventional white light and narrow band imaging revealed yellowish multinodular protrusions, in which irregular tortuous or dotted vessels were observed. Unexpectedly, biopsy of the lesion resulted in persistent bleeding. The biopsy specimen showed a large amount of amyloid deposition and calcification directly under the bronchial epithelium, as well as amyloid deposits in the blood vessel walls. In patients suspected to have amyloidosis, the presence of yellowish multinodular endobronchial protrusions, particularly with irregular vascularity, should prompt careful attention to avoid fatal postprocedural bleeding.

## Key points

In patients suspected to have amyloidosis, the possibility of bleeding should be considered and paid attention during biopsy of the affected organs. The presence of yellowish endobronchial multinodular protrusions with irregular vascularity may provide a predictive clue on persistent bleeding after biopsy.

## Introduction

Immunoglobulin light chain (AL) amyloidosis results from a small number of abnormal monoclonal plasma cells,^1^ which produce amyloid deposits that can damage the function of affected visceral organs. AL amyloidosis had been shown to increase the risk of bleeding, the mechanisms of which include vessel wall fragility, platelet dysfunction, and coagulation factor deficiency.^2^ A retrospective review of the literature revealed that 20 of 107 (24.3%) cases of tracheobronchial amyloidosis had bronchoscopic features with a propensity to bleed, even on touch.^3^ A definitive diagnosis of amyloidosis requires tissue biopsy. Although liver biopsy has been known to be complicated by life‐threatening bleeding, little is known about the risks of biopsy of other affected organs, such as transbronchial biopsy.^4^ Another recent review showed that the vascularization of the tissue samples was a significant risk factor for bleeding after bronchoscopic biopsy; for example, carcinoid tumor, endobronchial metastasis of renal cell carcinoma or thyroid cancer, and amyloidosis were prone to massive hemorrhage.^5^ However, there has so far been no detailed study regarding the association of the vascularization of amyloid tumors with iatrogenic bleeding during bronchoscopy. Here, we report the bronchoscopic findings on conventional white light and narrow band imaging (NBI) in a patient with amyloid endobronchial protrusions that bled easily.

## Case report

A 60‐year‐old man with no history of inflammatory diseases presented with multiple intrathoracic tumors. He had previously developed a left parotid tumor which had been diagnosed as parotid amyloidosis at another hospital. He reported a productive cough one week before the initial visit to our department, but denied any symptoms of fever, weight loss, night sweat, hemoptysis, chest pain, and shortness of breath. The remainder of his systems review was negative. Past medical and family history were not significant. He had smoked four cigarettes per day for 40 years. On examination, his respiratory rate was 12 breaths/min with oxygen saturation of 98% on room air, and a 5 cm subcutaneous tumor was palpable in the left parotid area. Breath sounds were decreased in both lower lung fields, although no crackles and wheezes were heard. Other physical examinations were normal.

During an eight month period of parotid tumor enlargement, the serum free kappa chain was elevated, ranging from 129 to 188 mg/L (reference, <19.4 mg/L), with an increase in the kappa/lambda ratio, ranging from 3.93 to 6.81 (reference, 0.26–1.65). Serum protein electrophoresis did not detect intact monoclonal immunoglobulin and bone marrow aspiration did not demonstrate the presence of myeloma. The levels of serum amyloid A and C‐reactive protein were not elevated. These findings suggested that the patient had primary AL amyloidosis. Circulating platelet count, prothrombin time, and partial thromboplastic time were normal. The levels of serum brain natriuretic peptide (BNP) and creatinine were not increased, and proteinuria was also not detected. The electrocardiogram showed normal sinus rhythm.

**Figure 1:**
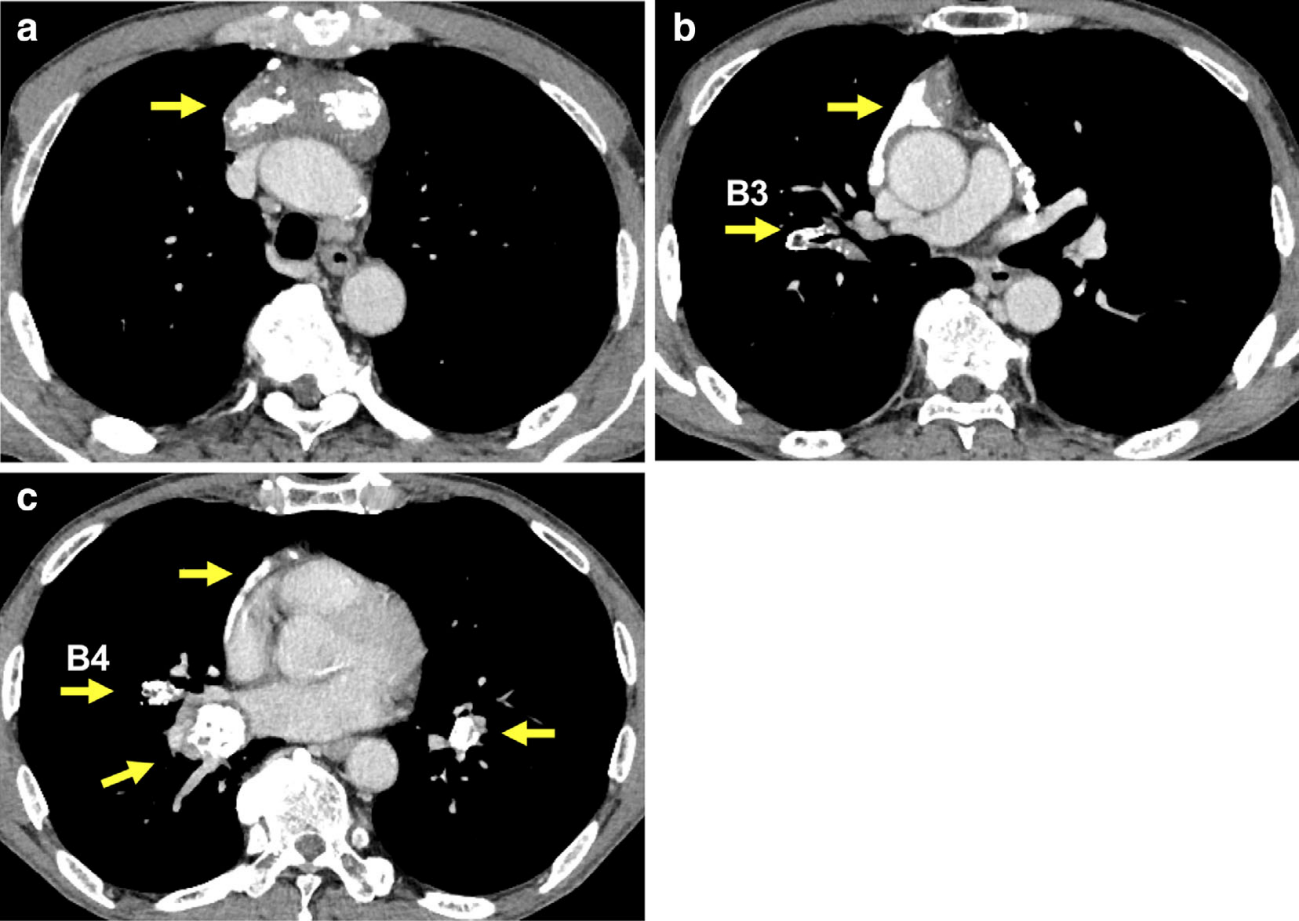
Contrast‐enhanced computed tomography images of the chest. There are markedly calcified tumors (arrows) in the **(a)** superior anterior mediastinum; **(b)** surrounding areas of the right B3 bronchus and the prevascular mediastinum; and **(c)** peribronchial area of the right B4 bronchus and the bilateral lower lobe bronchi.

**Figure 2:**
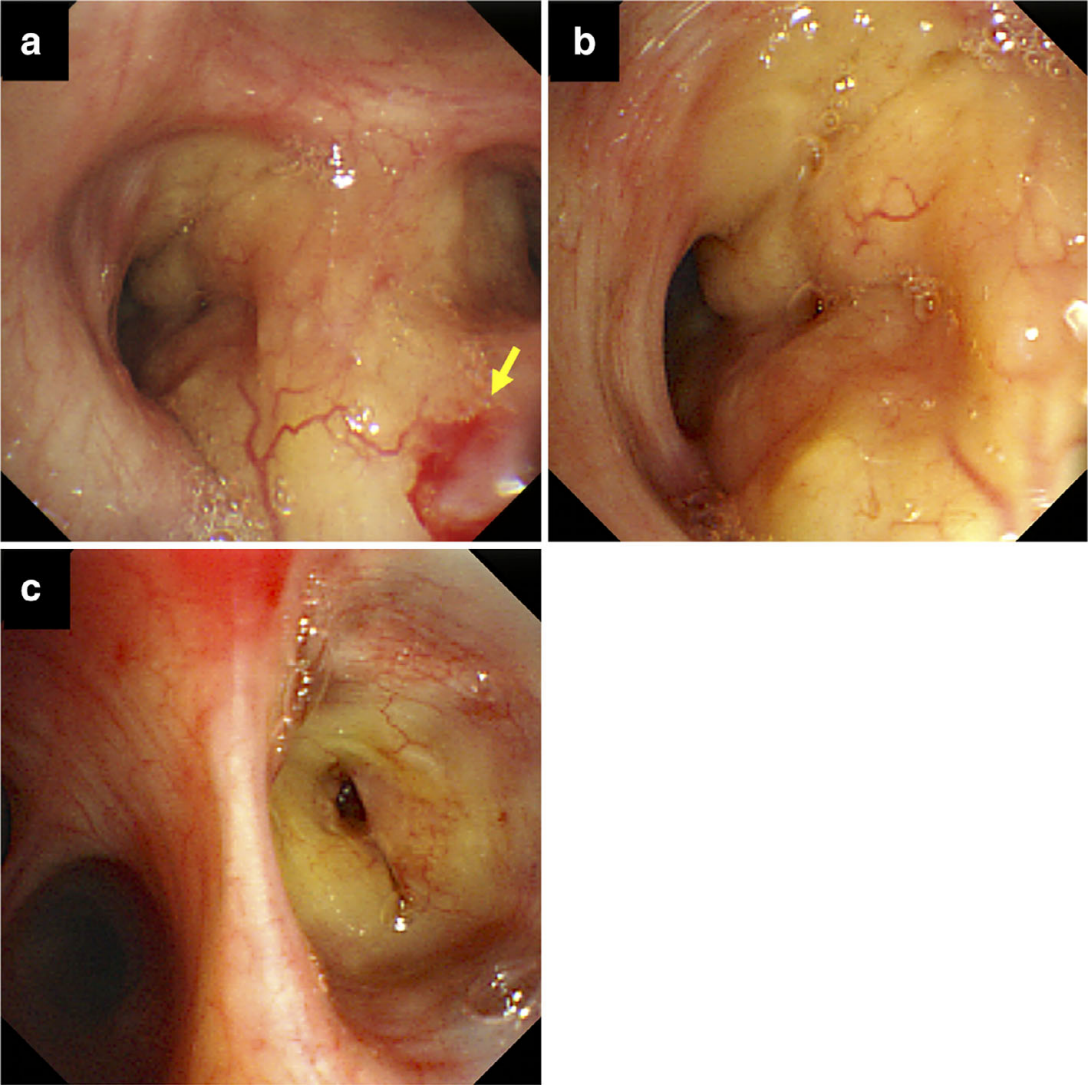
Bronchoscopic images. The mucosa had yellowish multinodular protrusions with tortuous vessels. **(a)** The right upper lobe bronchus shows spontaneous submucosal bleeding (arrow). The **(b)** right B3 bronchus and **(c)** right B4 bronchus show the tortuous superficial vessels.

**Figure 3:**
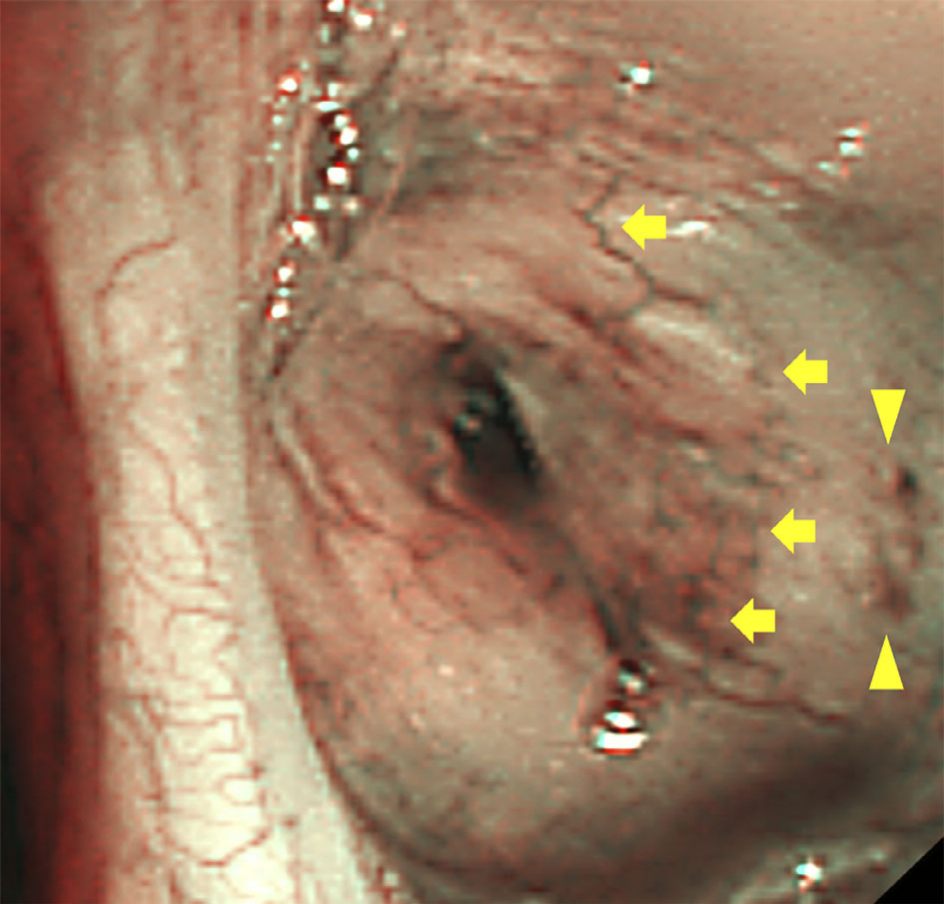
Narrow band imaging. The right B4 bronchus reveals irregular vascularity of the superficial vessels (brown) and deeper vessels (blue), with tortuosity (arrows) and dots (arrowheads).

Chest computed tomography scan showed markedly calcified solid tumors, without contrast enhancement, in the anterior mediastinum and bilateral peribronchial areas (Fig 1). Since the possibility of metastatic thymic neoplasms could not be completely excluded, bronchoscopic examination was performed. Conventional white light bronchoscopy revealed yellowish multinodular protrusions with tortuous vessels and spontaneous submucosal bleeding on the right B2, B3, B4, and bilateral lower lobe bronchi (Figs 2a–c). NBI bronchoscopy (BF‐1T260, Olympus, Tokyo, Japan) identified tortuous superficial and deeper vessels and dotted superficial vessels on the right B4 bronchus (Fig 3). Biopsy was performed on the protrusions on the right B4 bronchus and led to unexpected persistent bleeding. Finally, continuous suction by bronchoscopy successfully avoided serious bleeding.

**Figure 4:**
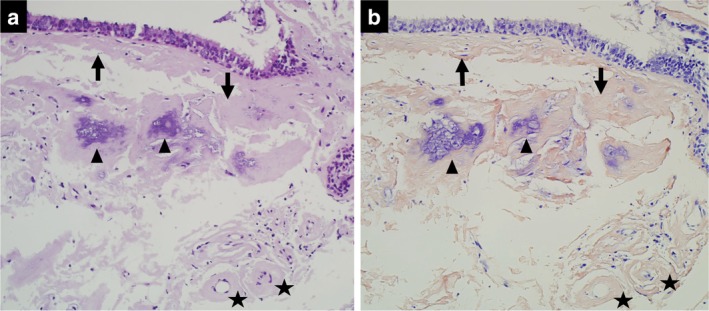
Photomicrographs of the biopsy specimen. **(a)** Hematoxylin and Eosin and **(b**) Congo red stains show amyloid deposition (arrows) and calcification (arrowheads) directly under the bronchial epithelium. Amyloid deposition is also seen in the blood vessel walls (stars) (each original magnification ×20).

On histology, the biopsy specimen demonstrated amyloid deposition and calcification directly under the epithelium, as well as amyloid deposits in the blood vessel walls (Fig 4). Amyloid protein was confirmed by Congo red stain combined with polarized light. Written informed consent for the publication of this case report was obtained from the patient.

## Discussion

The present case report provided two important findings, particularly the fact that amyloid endobronchial lesions that can easily bleed may appear as yellowish and multinodular protrusions on conventional white light bronchoscopy, with irregular subepithelial vascularity within the protrusion on NBI.

The mucosal appearance of bronchial amyloidosis tends to be yellowish in color and bleeds easily.^6^ Conventional white light bronchoscopy can penetrate the epithelium, as far as the subepithelial layer to a depth of 0.5 mm.^7^ Therefore, lesions inside the smooth muscle layer can be observed by conventional bronchoscopy. In the present case, the large amount of amyloid deposited directly under the bronchial epithelium probably explains the yellowish color of the protrusion. In the small intestine, multinodular protrusions probably represent massive amyloid deposition.^8^, ^9^ The mucosal appearance varies with the amount and distance of amyloid deposition from the epithelium.^8^, ^9^ Therefore, a yellowish color and multinodular feature of bronchial amyloidosis might provide a clue on bleeding tendency, although tracheobronchopathia osteochondroplastica, adenoid cystic carcinoma, and sarcoidosis should be considered as a differential diagnosis of yellowish multinodular lesions.^6^

Bronchoscopy under conventional white light showed a portion of the tortuous superficial vessels. On the other hand, NBI bronchoscopy enhanced the visibility of both tortuous deeper and superficial vessels. This was because the specific light wavelengths of NBI bronchoscopy were able to penetrate the tissue and were highly absorbed by hemoglobin. In a previous report on two patients with bronchial amyloid lesions, NBI bronchoscopy revealed capillary loops, tortuous vessels, and abrupt‐ending vessels but not dotted vessels; moreover, there was no mention of bleeding following a biopsy procedure.^10^ Our case demonstrated the presence of both dotted and tortuous vessels, which had been believed to result from irregular angiogenesis.^11^, ^12^, ^13^ However, the process of formation of irregular vascularity in the amyloid lesions remains uncertain.

In the present case, amyloid was deposited in the blood vessel walls. The majority of cases of bleeding in patients with amyloidosis has been attributed to amyloid deposition in the blood vessel walls.^14^ An experimental study showed that amyloid deposition induced oxidative stress, leading to the dysfunction and apoptosis of endothelial cells.^15^ Moreover, amyloid deposition can cause blood vessel fragility.^4^ Further studies are required to clarify the relationship between amyloid deposition in the blood vessels and bleeding tendency.

In conclusion, to the best of our knowledge, this was the first report to show both the white light and NBI bronchoscopic findings of easy bleeding amyloid endobronchial protrusions. Physicians should be aware that biopsy of yellowish multinodular protrusions, especially those with irregular subepithelial vascularity, may induce fatal postprocedural bleeding in patients suspected of amyloidosis.

## Disclosure

No authors report any conflict of interest.
